# Views of new internal medicine faculty of their preparedness and competence in physician-patient communication

**DOI:** 10.1186/1472-6920-6-30

**Published:** 2006-05-26

**Authors:** Paul S Mueller, Patricia A Barrier, Timothy G Call, Alan K Duncan, Daniel L Hurley, Adamarie Multari, Jeffrey T Rabatin, James TC Li

**Affiliations:** 1 From the Program in Professionalism, Department of Internal Medicine, Mayo Clinic, Rochester, Minnesota, USA; 2 Department of Internal Medicine, Mayo Clinic, 200 First Street SW, Rochester, MN 55905, USA

## Abstract

**Background:**

We sought to assess self-rated importance of the medical interview to clinical practice and competence in physician-patient communication among new internal medicine faculty at an academic medical center.

**Methods:**

Since 2001, new internal medicine faculty at the Mayo Clinic College of Medicine (Rochester, Minnesota) have completed a survey on physician-patient communication. The survey asks the new faculty to rate their overall competence in medical interviewing, the importance of the medical interview to their practice, their confidence and adequacy of previous training in handling eight frequently encountered challenging communication scenarios, and whether they would benefit from additional communication training.

**Results:**

Between 2001 and 2004, 75 general internists and internal medicine subspecialists were appointed to the faculty, and of these, 58 (77%) completed the survey. The faculty rated (on a 10-point scale) the importance of the medical interview higher than their competence in interviewing; this difference was significant (average ± SD, 9.4 ± 1.0 vs 7.7 ± 1.2, *P *< .001). Similar results were obtained by sex, age, specialty, years since residency or fellowship training, and perceived benefit of training. Experienced faculty rated their competence in medical interviewing and the importance of the medical interview higher than recent graduates (ie, less than one year since training). For each challenging communication scenario, the new faculty rated the adequacy of their previous training in handling the scenario relatively low. A majority (57%) said they would benefit from additional communication training.

**Conclusion:**

Although new internal medicine faculty rate high the importance of the medical interview, they rate their competence and adequacy of previous training in medical interviewing relatively low, and many indicate that they would benefit from additional communication training. These results should encourage academic medical centers to make curricula in physician-patient communication available to their faculty members because many of them not only care for patients, but also teach clinical skills, including communication skills, to trainees.

## Background

Medical interviewing is one of the most important skills in medicine and effective physician-patient communication is essential for optimal medical care. Effective physician-patient communication is associated with improved patient satisfaction [1-6], increased patient adherence with medications and recommendations [1,6-8], improved medical outcomes [6,7,9], and less malpractice risk [10]. Indeed, consensus statements [11,12] have concluded that effective physician-patient communication is an integral part of medical practice. The Association of American Medical Colleges (AAMC) [13] and the American College of Physicians [14] regard effective communication as a key attribute of professionalism, and the Accreditation Council for Graduate Medical Education (ACGME) [15] describes effective interpersonal and communication skills as a general competency common to all specialties.

Although previous surveys [16-20] assessed preparedness and competence in communication among internists, they did so only partially (eg, one or two questions). In addition, these surveys did not assess the preparedness for specific physician-patient communication scenarios. Herein, we describe the results of a survey that assessed self-rated preparedness, competence, and perceived benefit of additional training in physician-patient communication among new internal medicine faculty, regardless of their age or previous experience, at an academic medical center.

## Methods

Since 2001, the Department of Internal Medicine of the Mayo Clinic College of Medicine (Rochester, Minnesota) has conducted a physician-patient communication program for new faculty members. In an effort to assess self-rated importance of the medical interview to clinical practice and competence in physician-patient communication and perceived benefit of additional physician-patient communication training, the new faculty members were asked to complete a survey. The survey asked new faculty to report sex, age, specialty, years since completing training, and whether they would benefit from additional communication training ("yes," "no," or "maybe"). Using 10-point Likert scale questions, the survey also asked the faculty to rate their overall competence in medical interviewing, the importance of the medical interview to their practices, and their confidence and adequacy of previous training (eg, during medical school and residency) in handling eight frequently encountered challenging physician-patient communication scenarios. The survey questions are listed in Table 1. The survey was conducted during the years 2001 through 2004. The results of the survey comprise the data set of this study.

### Statistical analysis

For within-participant comparisons of continuous outcomes, Wilcoxon signed rank tests were used. The relation between continuous outcomes was modeled using an ordinary least squares fit of a linear model, and *F*-tests were used for a test of association. For between-participant group comparisons of continuous outcomes, Wilcoxon rank sum tests were used. To test for independence of variables (eg, sex, age, specialty, and years since completing training), an analysis of variance (ANOVA) was done where feasible and appropriate. Significance was a *P *value of .05 or less. All analyses were conducted using JMP 4.0.4 (SAS Institute, Inc., Cary, North Carolina). Permission to perform an analysis of the surveys was granted by the Mayo Foundation Institutional Review Board in accordance with federal regulations.

## Results

Between 2001 and 2004, 75 general internists and internal medicine subspecialists were appointed to the faculty of the Mayo Clinic College of Medicine (Rochester, Minnesota). Of these, 58 (77%) completed the survey. Of the 58 survey respondents, 37 (64%) were men, 43 (75%) were 40 years old or younger, 40 (69%) were subspecialists, and 22 (39%) were recent (within 1 year) graduates of a residency or fellowship training program. Thirty-three participants (57%) said they would benefit from additional communication training, 24 (41%) said they might benefit, and one (2%) said he or she would not benefit (Table 2).

On a 10-point scale, the average ± standard deviation of self-rated competence (1 = not competent, 10 = highly competent) in medical interviewing by the new faculty was 7.7 ± 1.2. General internists rated themselves more competent in medical interviewing than subspecialists did (8.1 ± 1.1 vs 7.5 ± 1.1, *P *= .042), as did faculty who had completed their residency or fellowship training more than one year before beginning the physician-patient communication curriculum compared with recent graduates (7.9 ± 1.0 vs 7.3 ± 1.4, *P *= .032). There were no differences in self-rated competence in medical interviewing according to sex, age, or perception of benefiting from the curriculum (Table 2). On the basis of these results and the limited sample size, we performed ANOVA using the variables sex, specialty, and years since training. After accounting for sex and years since training, a statistically significant association was not found between specialty (general internist vs subspecialist) and self-rated competence in medical interviewing (*P *= .162), and a marginally significant association was found between years since training (ie, more than one year) and self-rated competence (*P *= .055).

The average self-rated importance of the medical interview in the practices of the new faculty was 9.4 ± 1.0. Faculty who had completed their residency or fellowship training more than one year before beginning the curriculum rated the importance of the medical interview higher than did recent graduates (9.6 ± 0.6 vs 9.0 ± 1.3, *P *= .039). There were no statistically significant differences in self-rated importance of the medical interview according to sex, age, specialty, or perception of benefiting from the curriculum (Table 2). ANOVA was then performed using the variables sex, specialty, and years since training. After accounting for sex and specialty, a statistically significant association between years since training (ie, more than one year) and self-rated importance of the medical interview was present (*P *= .012).

New faculty rated their overall competence in interviewing lower than the importance of the medical interview to practice. Matched-pairs testing revealed a significant difference when comparing how faculty rated their overall competence in medical interviewing and how they rated the importance of the medical interview in practice (difference 1.7 [95% CI, 1.4–2.0], *P *< .001). Similar results were obtained by sex, age, generalist versus specialist, years since training, and perceived benefit of training (Table 2).

The new faculty were asked to rate their confidence and adequacy of their previous training in handling eight frequently encountered challenging physician-patient communication scenarios. For every scenario, the new faculty rated the adequacy of their previous training in handling the scenarios relatively low (range, 4.5 ± 2.4 to 6.1 ± 2.4). Furthermore, for each scenario, new faculty rated their confidence in handling the scenario higher than their adequacy of training. Indeed, matched-pairs testing showed significant differences in self-rated confidence in handling and self-rated adequacy of training for every scenario. For each scenario, a significant percentage of variability of self-rated confidence in handling the scenario was associated with self-rated adequacy of previous training (Table 3). Similar results were obtained when the data were analyzed by subgroup (ie, sex, age, generalist vs specialist, and years since training) (data not shown).

The new faculty who said they would benefit from additional communication training rated the adequacy of their previous training in handling six of the challenging communication scenarios significantly lower than the faculty who said they might or would not benefit from communication training (Table 4).

## Discussion

Several previous surveys have partially assessed preparedness and competence in physician-patient communication. For example, a survey of 210 graduates of an internal medicine training program rated the adequacy of their communication training significantly lower than the importance of those skills to clinical practice [16]. Several other surveys of graduates of internal medicine training programs had similar results [17-19]. Finally, in a telephone survey of 300 physicians, 92% rated effective communication as important; yet, 28% rated the adequacy of their training in physician-patient communication as fair or poor [20].

The results of our survey add to those of previous surveys in several important ways. First, whereas the previous surveys partially assessed physician-patient communication (eg, only one or two questions), our survey was focused entirely on this topic. Second, unlike previous surveys, ours focused on new internal medicine faculty at an academic medical center regardless of their age or previous experience. Third, we not only assessed self-rated importance of the medical interview to clinical practice and competence in medical interviewing among the new faculty, we also assessed self-rated confidence and perceived adequacy of previous training in 8 challenging physician-patient communication scenarios. Fourth, we specifically asked the new faculty whether they would benefit from additional training in physician-patient communication. Finally, unlike previous surveys, we analyzed our results according to sex, age, years in practice, and generalist versus subspecialty focus.

Our new internal medicine faculty rated the importance of medical interviewing relatively high. This high regard for medical interviewing is consistent with consensus statements [11,12] that concluded effective physician-patient communication is an integral part of medical practice and the position of the ACGME that effective communication is a general competency important to all specialties [15]. However, our faculty also rated the importance of medical interviewing to clinical practice significantly higher than their competence in medical interviewing. There are several possible reasons for this difference. On the one hand, the difference may be true. The statistical analysis (matched pairs) compared the means of 2 related medical interviewing topics – self-rated importance of and competence in medical interviewing – from the same group of physicians. A higher rating for importance than for competence suggests a perceived educational gap. On the other hand, the difference may not be true, but rather reflect different scales of judgment. Although a 10-point scale was used for all the survey questions, directly comparing self-rated importance of with self-rated competence in medical interviewing assumes the scales themselves are comparable (ie, each particular value on the importance scale means the same on the competence scale). In other words, physicians may naturally rate the importance of medical interviewing higher than their competence in interviewing not because of a true difference but because the items being rated are different (ie, akin to comparing an apple to an orange).

Older (age >40 years) and more experienced new faculty (completed residency or fellowship training more than one year before beginning the communication curriculum) rated the importance of medical interviewing higher than younger new faculty and those who were recent graduates. One reason for these differences may be the relative lack of clinical experience (compared with that of more experienced physicians) among younger faculty and recent graduates for whom the value and centrality of the medical interview to clinical practice has not been fully realized. Another reason may be that physician-patient communication is not part of the formal curriculum, and trainees may graduate from their program with the conception that effective physician-patient communication is not as important as other skills. Indeed, training in communication skills is underemphasized during internal medicine residency training [16,17,19]. Finally, we found that more experienced new faculty rated their competence in medical interviewing higher than less experienced faculty. Over time, it is likely that physicians expand their repertoires of communication skills and competencies in using them. Indeed, communication skills may improve with time and experience alone [21].

Our new faculty rated the adequacy of their previous training in handling 8 challenging physician-patient communication scenarios relatively low. For each scenario, a significant percentage of variability of confidence in handling the scenario was associated with self-rated adequacy of previous training (Table 3). Although it is plausible that confidence in handling the scenarios is influenced by previous training, our study does not establish a causal relationship. An alternative explanation may be that confidence in handling the scenarios is influenced by learning and that the less confident faculty attributed their confidence to their previous training programs rather than their own learning.

Importantly, a majority (57%) of our new faculty specifically said they would benefit from additional training in physician-patient communication. These faculty also rated the adequacy of their training in handling six of the challenging physician-patient communication scenarios significantly lower than those who said they might or would not benefit from additional training (Table 4). These findings are important because many internal medicine faculty at academic medical centers not only care for patients, but also teach clinical skills, including communication skills, to medical students, residents, and other trainees [22]. Teaching these skills, however, requires qualified and willing faculty members [23].

Our results also suggest that many new internal medicine faculty may perceive a need for additional communication training. Such training may enhance clinical practice and teaching and role-modeling communication skills to trainees. In fact, evidence suggests that effective physician-patient communication skills can be taught and learned [24]. Furthermore, training may improve medical outcomes (eg, improving patient satisfaction and reducing patient emotional distress) [25-27]. A review of specific curricula that might be used to teach such skills is beyond the scope of this paper.

Our survey has a number of limitations. First, the results may not apply to institutions unlike ours and to physicians who are not general internists or internal medicine specialists. Second, the survey used data derived from self-reported ratings of physician competence, confidence, and adequacy of previous training in communication (not measures of actual competency such as assessments of actual interactions between physicians and patients). Also, they may be influenced by recall bias [16] and experiences during the time since training [18]. However, a previous study of physicians found that performance correlated with self-reported preparedness for clinical practice [28]. Third, the size of our cohort was relatively small. However, this size was similar to that of some of the studies discussed above.

## Conclusion

Although new internal medicine faculty regard medical interviewing as important to clinical practice, they also rate the adequacy of their training in handling challenging communication scenarios relatively low. In fact, many faculty specifically stated they would benefit from additional training in physician-patient communication. These results should encourage academic medical centers to make curricula (ie, faculty development) in physician-patient communication available to their faculty because many of them not only care for patients, but also teach clinical skills, including communication skills, to trainees.

## Competing interests

The author(s) declare that they have no competing interests.

## Authors' contributions

All authors have given final approval of the submitted manuscript.

The following authors have participated in the work and take responsibility for part of the content (P.A.B., T.G.C., A.K.D., D.L.H., A.M., J.T.R., and J.T.C.L.), the whole content (P.S.M.), contributed to conception and design (P.S.M., P.A.B., T.G.C., A.K.D., D.L.H., A.M., J.T.R., and J.T.C.L.), acquisition of data (P.S.M., P.A.B., T.G.C., A.K.D., D.L.H., A.M., J.T.R., and J.T.C.L.), analysis and interpretation of data (P.S.M., J.T.R., and J.T.C.L.), drafting of the manuscript (P.S.M.), and critical revision of the manuscript for important intellectual content (P.S.M., P.A.B., T.G.C., A.K.D., D.L.H., A.M., J.T.R., and J.T.C.L). The Mayo Clinic (Rochester, Minnesota) Center for Patient-Oriented Research provided statistical support.

## Pre-publication history

The pre-publication history for this paper can be accessed here:



## Figures and Tables

**Table 1:** Survey of New Internal Medicine Faculty at Mayo Clinic to Assess Self-Rated Competency and Adequacy of Training in Physician-Patient Communication*

**Survey questions**

1. Please rate your overall competence in medical interviewing (1 = not competent, 10 = highly competent)
2. How important is the medical interview in your practice? (1 = not important, 10 = very important)
3. How confident are you in your ability to successfully handle communication in each of the following scenarios? (1 = not confident, 10 = very confident)
4. How adequate was the training you received during medical school, residency, and/or fellowship in preparing you to successfully handle communication in each of the following scenarios? (1 = inadequate, 10 = excellent)
**Scenarios**
a. The patient who makes excessive demands for tests and/or treatments
b. Counseling the overweight patient with medical complications due to obesity for whom treatments of obesity failed in the past
c. Informing a young woman with a new breast mass that the biopsy shows cancer
d. A patient's spouse who is demanding and hostile during the interview
e. The patient with somatoform disorder who presents with a long list of problems and extensive medical records
f. The patient with chronic pain who is taking multiple narcotics and refuses psychiatric evaluation
g. The patient who has been kept waiting an hour or more beyond the appointment time
h. The patient whose cultural background is significantly different from yours

*Questions answered with a 10-point Likert scale.

**Table 2:** Demographic Features, Self-Rated Competence in Medical Interviewing, and Importance of the Medical Interview to Practice for 58 New Internal Medicine Faculty

Characteristics	No. of faculty	Self-rated competence in medical interviewing, score, average ± SD*	Self-rated importance of medical interview to practice, score, average ± SD*	Matched pairs mean difference between self-rated importance of medical interview and competence in interviewing (95% CI)	*P *value^†^
All participants	58	7.7 ± 1.2	9.4 ± 1.0	1.7 (1.4–2.0)	<.001
Sex					
Male	37	7.5 ± 1.2	9.2 ± 1.1	1.7 (1.3–2.1)	<.001
Female	21	8.0 ± 1.0	9.6 ± 0.6	1.6 (1.1–2.2)	<.001
*P *value^‡^		.115	.180		
Age, y					
≤ 40	43	7.7 ± 1.0	9.2 ± 1.0	1.5 (1.1–1.8)	<.001
>40	14	7.5 ± 1.6	9.8 ± 0.6	2.3 (1.5–3.0)	<.001
*P *value^‡^		.870	.059		
Specialty					
Generalist	18	8.1 ± 1.1	9.5 ± 0.9	1.4 (1.0–1.8)	<.001
Specialist	40	7.5 ± 1.1	9.3 ± 1.0	1.8 (1.4–2.2)	<.001
*P *value^‡^		.042^§^	.293		
Years since training^//^					
≤ 1	22	7.3 ± 1.4	9.0 ± 1.3	1.7 (1.0–2.3)	<.001
>1	35	7.9 ± 1.0	9.6 ± 0.6	1.6 (1.3–2.0)	<.001
*P *value^‡^		.032	.039		
Benefit from additional communication training?					
Yes	33	7.5 ± 1.2	9.4 ± 0.8	1.9 (1.5–2.3)	<.001
Maybe or no^¶^	25	8.0 ± 1.1	9.3 ± 1.1	1.4 (0.8–1.9)	<.001
*P *value^‡^		.162	.860		

*Ten-point Likert scale (eg, 1 = not competent, 10 = highly competent).

^†^Wilcoxon signed rank test.

^‡^Wilcoxon rank sum test.

^§^Not significant after ANOVA (see text).

//One participant did not indicate age or years since training.

^¶^Twenty-four participants answered "maybe" and 1 answered "no."

**Table 3:** Self-Rated Confidence Versus Adequacy of Previous Training in Handling Various Physician-Patient Communication Scenarios of New Faculty Beginning Communication Curriculum

Physician-patient communication scenario	Score, average ± SD*	Matched pairs mean difference confidence vs adequacy of previous training (95% CI)	*P *value^†^	Percent variability of C explained by A	*P *value^‡^
Demanding patient					
C	6.9 ± 1.8	1.7 (1.2–2.2)	<.001	42	<.001
A	5.2 ± 2.5				
Overweight patient					
C	6.7 ± 1.9	1.9 (1.2–2.5)	<.001	23	<.001
A	4.8 ± 2.3				
Cancer patient					
C	7.6 ± 1.8	1.4 (0.7–2.0)	<.001	17	.006
A	6.1 ± 2.4				
Hostile spouse					
C	6.7 ± 1.7	2.2 (1.6–2.8)	<.001	20	<.001
A	4.5 ± 2.4				
Somatoform patient					
C	6.7 ± 1.8	1.7 (1.1–2.3)	<.001	24	<.001
A	5.0 ± 2.5				
Chronic pain patient					
C	6.2 ± 1.9	1.5 (0.8–2.1)	<.001	21	<.001
A	4.8 ± 2.5				
Waiting patient					
C	7.4 ± 1.5	2.5 (1.9–3.2)	<.001	18	.007
A	4.9 ± 2.5				
Patient of different culture					
C	7.3 ± 1.7	2.0 (1.3–2.7)	<.001	14	.024
A	5.0 ± 2.4				

A, self-rated adequacy of previous training in handling the given physician-patient communication scenario; C, self-rated confidence in handling the given physician-patient communication scenario; CI, confidence interval; SD, standard deviation.

*Ten-point Likert scale (C: 1 = not confident, 10 = very confident; A: 1 = inadequate, 10 = excellent).

^†^Wilcoxon signed rank test.

^‡^Bivariate fit of self-rated confidence by self-rated adequacy of previous training.

**Table 4:** Self-Rated Adequacy of Previous Training in Handling Specific Physician-Patient Communication Scenarios According to Perceived Benefit of Training

	Self-rated adequacy of previous training* according to perceived benefit of training		
		
Scenario	Yes (N = 33)	Maybe or no (N = 25)	*P *value^†^
Demanding patient	4.4 ± 2.4	6.2 ± 2.4	.006
Overweight patient	4.2 ± 2.2	5.6 ± 2.2	.028
Cancer patient	5.7 ± 2.5	6.7 ± 2.2	.078
Hostile spouse	3.7 ± 2.4	5.5 ± 2.1	.002
Somatoform patient	4.2 ± 2.4	6.1 ± 2.3	.004
Chronic pain patient	4.0 ± 2.3	5.8 ± 2.4	.007
Waiting patient	4.7 ± 2.6	6.1 ± 2.7	.002
Patient of different culture	4.2 ± 2.1	6.2 ± 2.3	.067

*Ten-point Likert scale (1 = inadequate, 10 = excellent).

^†^Wilcoxon rank sum test.
